# Genetic characterization and molecular identification of the bloodmeal sources of the potential bluetongue vector *Culicoides obsoletus* in the Canary Islands, Spain

**DOI:** 10.1186/1756-3305-5-147

**Published:** 2012-07-24

**Authors:** Josué Martínez-de la Puente, Javier Martínez, Martina Ferraguti, Antonio Morales-de la Nuez, Noemí Castro, Jordi Figuerola

**Affiliations:** 1 Departamento de Ecología de Humedales, Estación Biológica de Doñana (EBD-CSIC), C/Américo Vespucio, s/n, Seville E-41092, Spain; 2 Departamento de Microbiología y Parasitología, Facultad de Farmacia, Universidad de Alcalá, Alcalá de Henares, Madrid E-28871, Spain; 3 Department of Animal Science, Universidad de las Palmas de Gran Canaria, Arucas E-35413, Spain

**Keywords:** Bluetongue virus, Bloodmeal, *Culicoides*, Goats, Schmallenberg virus, Sheep

## Abstract

**Background:**

*Culicoides* (Diptera: Ceratopogonidae) biting midges are vectors for a diversity of pathogens including bluetongue virus (BTV) that generate important economic losses. BTV has expanded its range in recent decades, probably due to the expansion of its main vector and the presence of other autochthonous competent vectors. Although the Canary Islands are still free of bluetongue disease (BTD), Spain and Europe have had to face up to a spread of bluetongue with disastrous consequences. Therefore, it is essential to identify the distribution of biting midges and understand their feeding patterns in areas susceptible to BTD. To that end, we captured biting midges on two farms in the Canary Islands (i) to identify the midge species in question and characterize their COI barcoding region and (ii) to ascertain the source of their bloodmeals using molecular tools.

**Methods:**

Biting midges were captured using CDC traps baited with a 4-W blacklight (UV) bulb on Gran Canaria and on Tenerife. Biting midges were quantified and identified according to their wing patterns. A 688 bp segment of the mitochondrial COI gene of 20 biting midges (11 from Gran Canaria and 9 from Tenerife) were PCR amplified using the primers LCO1490 and HCO2198. Moreover, after selected all available females showing any rest of blood in their abdomen, a nested-PCR approach was used to amplify a fragment of the COI gene from vertebrate DNA contained in bloodmeals. The origin of bloodmeals was identified by comparison with the nucleotide-nucleotide basic alignment search tool (BLAST).

**Results:**

The morphological identification of 491 female biting midges revealed the presence of a single morphospecies belonging to the *Obsoletus* group. When sequencing the barcoding region of the 20 females used to check genetic variability, we identified two haplotypes differing in a single base. Comparison analysis using the nucleotide-nucleotide basic alignment search tool (BLAST) showed that both haplotypes belong to *Culicoides obsoletus*, a potential BTV vector. As well, using molecular tools we identified the feeding sources of 136 biting midges and were able to confirm that *C. obsoletus* females feed on goats and sheep on both islands.

**Conclusions:**

These results confirm that the feeding pattern of *C. obsoletus* is a potentially important factor in BTV transmission to susceptible hosts in case of introduction into the archipelago. Consequently, in the Canary Islands it is essential to maintain vigilance of *Culicoides-*transmitted viruses such as BTV and the novel Schmallenberg virus.

## Background

Biting midges of the genus *Culicoides* Latreille (Diptera: Ceratopogonidae) comprise a highly diverse biological group with more than 1,400 species worldwide [1]. Many biting midge species have ecological [2], economic [3] and sanitary [4] relevance as haematophagous insects and as vectors of pathogens in humans, livestock, poultry and wildlife.

Bluetongue virus (BTV) is one of the most important disease agents transmitted by biting midge *Culicoides*[4,5]. Bluetongue disease (BTD) largely affects ruminants [5], above all in America, Australia, Asia and Africa, and in recent years has expanded into Europe. This spread is probably closely linked to a northward expansion of *Culicoides imicola* Kieffer, the main Afro-tropical vector species, and the ability of autochthonous Palaearctic *Culicoides* species to transmit BTV [1]. Among native *Culicoides* species, *C. obsoletus* Meigen has been identified as a potential vector of BTV by using different procedures including viral isolation and RT-PCR detection [6,7], as well as experimental infection assays [8]. In addition, this species is thought to have been involved in the outbreaks produced by different BTV serotypes that have occurred in recent years in Europe [9].

The morphological identification of female biting midges to species level is a complex task that usually requires great taxonomic expertise [10]. Their minute size and the huge number of existing biting midge species are two of the main reasons why field studies on *Culicoides* are laborious and have lagged behind those conducted on other insect vectors [4]. Wing patterns and the morphology of several parts of the body, which usually requires the dissection and separation of the wings, head, abdomen and genitalia, are the main characters employed in species identification [11]. Recently, the morphological identification of biting midges *Culicoides* has been complemented by the use of molecular tools and as a result more accurate species identification is now possible. In particular, the amplification of a fragment of the mitochondrial cytochrome oxidase subunit I (COI) gene is emerging as a useful and effective tool that can facilitate the identification of *Culicoides* species [12-15] and animals in general [16]. This procedure is especially useful when identifying females of sibling species within species complexes, which are difficult to identify using available morphological information; this is the case for a number of species in the *Culicoides obsoletus* species complex, which contains both *C. obsoletus* and *C. scoticus* Downes & Kettle.

**Figure 1:**
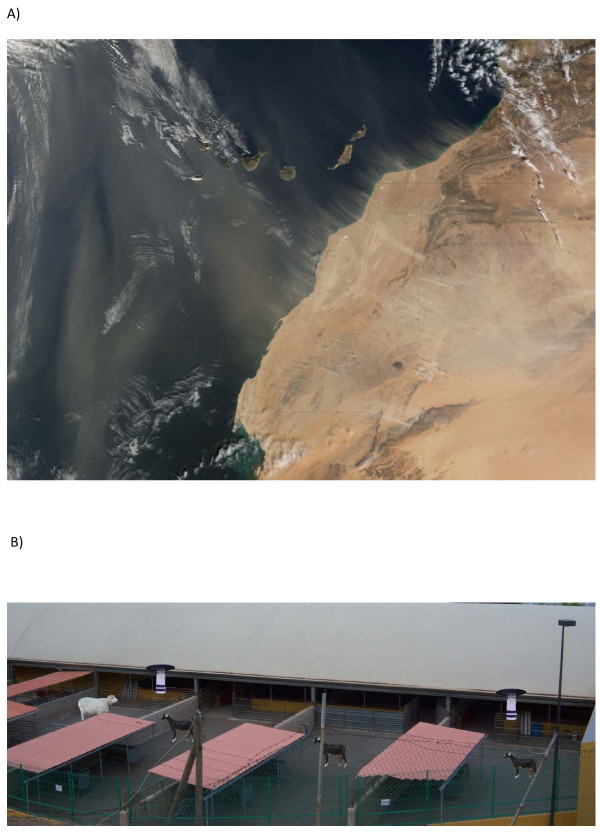
**a) Image taken on 20 January 2008 by the Moderate Resolution Imaging Spectroradiometer (MODIS) flying on NASA’s Terra satellite showing numerous plumes of dust blowing off the African coast to Canary Islands (accessible at:**http://visibleearth.nasa.gov/view.php?id=19565**).b**) Location of CDC-type downdraft miniature suction traps and the location of the livestock in the Gran Canaria farm. Note that the number of animals does not correspond with the actual number of animals during the study.

Located around 100 km off the African coast, the Canary Islands archipelago (Spain) has one of the highest densities of small ruminants per hectare of agricultural land in the whole of Europe. Although the relationship between ruminant density and the efficient spread of bluetongue is yet to be tested, animal density is thought to play an important role in the spread of this disease [17]. Despite being considered free of BTD [18], it is still important to improve our knowledge of the occurrence of biting midges on these islands. The arrival of the disease in hitherto BTD-free areas could be provoked by the movement of infected livestock or by the passive movement of infected *Culicoides* on winds, especially over the sea [19,20]. Winds transporting sand from the Sahara desert, known locally as *calimas* (Figure 1a), are habitual in the Canary Islands and potentially transport biting midges from localities in which they are present (see ref. [21]). In addition, *C. obsoletus* and *C. analis* Santos Abreu have both been previously reported in the Canary Islands [22]: *C. obsoletus* has been captured in Tenerife and La Palma [23] and, according to recent information provided by the Spanish National Surveillance Programme, *C. obsoletus* is present in Fuerteventura and Gran Canaria [18]. The aims of our study were: (i) to identify species and characterize the COI barcoding region of biting midges captured on these islands and (ii) to ascertain the source of bloodmeals from biting midge females using molecular tools that permit the identification of the feeding sources of the bluetongue vector *C. obsoletus* in the studied areas. We thus captured biting midges from two farms on the Canary Islands, one on Gran Canaria and the other on Tenerife.

## Methods

Biting midges were captured using two CDC-type downdraft miniature suction traps baited with a 4-W blacklight (UV) bulb (model 1212; J.W.Hock, Gainesville, FL) at a distance of 21 m. The light traps were operated from sunset to sunrise for 19 nights from 15/09/2010 to 15/12/2010 on the farm of the Universidad de Las Palmas de Gran Canaria (Figure 1b; 28°8′21 N, 15°30′24 W, Arucas, Gran Canaria). During this period, on two nights one trap only was operating at this site. Traps were hung 2.2 m above ground level and were operated with a UV light. UV light traps are regarded as useful surveillance tools for measuring the relative abundance of midges, although, it is possible that this procedure may not be a reliable indicator of species composition [24]. As well, for four nights from 05/12/2010 to 11/12/2010, a single trap was placed in the El Pico farm of the Instituto Canario de Investigaciones Agrarias (28°31′24 N, 16°22′19 W, Tegueste, Tenerife). All biting midges were preserved in ethanol and maintained at −80°C until further analysis.

All the biting midges collected were quantified and sorted according to their wing patterns [11] using an Olympus SZH stereomicroscope (10 × –64× magnification). Bloodfed females were individually stored for blood-source identification (see below).

### Molecular characterization of biting midges

Genomic DNA from 20 morphologically identified biting midges (11 from Gran Canaria and nine from Tenerife) was extracted as indicated in ref [2].

A 688 bp segment of the mitochondrial COI gene was PCR amplified from individual biting midges using the primers LCO1490 and HCO2198 ([25], see Table 1). The PCR reaction volume of 20 μL consisted of between 20 and 100 ng DNA template, 50 mM KCl, 10 mM Tris–HCl, 1.5 mM MgCl_2_, 0.05 mM of each dNTP, 0.5 μM of each primer and 1.25 U of AmpliTaq Gold 360 (Applied Biosystems, Foster City, CA, USA). The reactions were cycled using a Verity thermal cycler (Applied Biosystems) according to the following parameters: 94°C for 10 min (polymerase activation), 40 cycles at 95°C for 30 s (denaturing), 46°C for 30 s (annealing temperature), 72°C for 1 min (extension), and a final extension at 72°C for 10 min. Amplicons obtained after PCR assays were recovered from agarose gels, purified using the MoBio kit UltraClean GelSpin and subjected to direct sequencing. DNA fragments obtained were sequenced using an ABI 3130 (Applied Biosystems) automated sequencer.

**Table 1:** Primers used in this study to identify biting midges and the sources of their vertebrate bloodmeals

**Primer**	**Sequence (5' - > 3')**
*Biting midge identification*^a^	
LCO1490	GGTCAACAAATCATAAAGATATTGG
HCO2198	TAAACTTCAGGGTGACCAAAAAATCA
*Vertebrate identification*^b^	
BCFW-M13	TGTAAAACGACGGCCAGTHAAYCAYAARGAYATYGG
BCRV1	GCYCANACYATNCCYATRTA
M13	GTAAAACGACGGCCAGTG
BCRV2	ACYATNCCYATRTANCCRAANGG

^a^Primers previously published by [25].

^b^Primers previously published by [26].

### Bloodmeals identification

In all, 290 females (74 females from Gran Canaria and 216 from Tenerife) were identified as blood-fed. Using a conservative approach, we included all available females showing any rest of blood in their abdomen to detect the maximum number of potential hosts, thus we included fully engorged and partially engorged females and blood-fed biting midges that, due to the shape and colour of the abdomen, probably contained a partially digested bloodmeal. After excluding some blood-fed females that showed external remains of blood from other damaged blood-fed arthropods, 267 biting midges were processed to identify the origin of their bloodmeals. The abdomen of individual blood-fed biting midges was cut off using sterile tips and introduced into 50 μl of lysis solution (25 mM NaOH, 0.2 mM EDTA), crushed and incubated at 95°C for 30 min. At least two negative DNA extraction controls (i.e. absence of tissue) were performed during the PCR experiments. After incubation, the solution was cooled for five minutes, after which time 50 μl of neutralization solution (40 mM Tris–HCl) was added. Abdomens were simultaneously processed using 96-thermowell plates and stored at −20°C until PCR amplification. Bloodmeal sources were identified using the protocol described and tested in ref. [26] to amplify a fragment of the vertebrate COI gene (Table 1). This method is used to amplify DNA from blood of a diversity of vertebrate species in the abdomen of arthropods including mosquitoes and biting midges [26]. Briefly, this procedure used a nested-PCR approach, using the primary pair of primers M13BCV-FW and BCV-RV1 and the nested primer pair M13 and BCV-RV2. Sequences were edited using the software Sequencher v4.9 (Gene Codes, © 1991 - 2009, Ann Arbor, MI) and identified by comparison with the nucleotide-nucleotide basic alignment search tool (BLAST) (GenBank DNA sequence database, National Center for Biotechnology Information) to assign unknown COI sequences to particular vertebrate species. Host species assignment was considered completed when we found a match of 98% or more between our sequences and those in GenBank.

## Results

A total of 491 biting midge females (201 in Gran Canaria and 290 in Tenerife) and eight males (seven in Gran Canaria and one in Tenerife) were captured during the study. Based on wing patterns, all females were morphologically identified as belonging to the *C. obsoletus* complex. Amongst the 11 biting midge females from Gran Canaria and nine biting midge females from Tenerife we identified two haplotypes (haplotypes C1 and C2) that differed only in a single base. With the exception of a single biting midge from Gran Canaria, which was identified as C2, the rest of the biting midges analysed corresponded to the haplotype C1. These two haplotypes were unequivocally identified as *C. obsoletus* by comparison with the GenBank DNA sequence database. *Culicoides* body parts from three specimens (two C1 and one C2 haplotypes) not used in molecular analyses have been deposited in the collection of the Museo Nacional de Ciencias Naturales (MNCN-CSIC), Madrid, Spain (accession numbers: MNCN/ADN 17017, 17018 and 17019). As well, the sequences obtained from these biting midges have been deposited in GenBank [GenBank: JQ740594, GenBank: JQ740595, GenBank: JQ740596].

In all, we obtained positive PCR products from 136 blood-fed females. Biting midges were found to have fed on both goats and sheep in the Canary Islands: specifically, on Gran Canaria, 40 *Culicoides* females had fed on goats (*Capra hircus*) and 14 on sheep (*Ovis aries*), while on Tenerife, 81 females had fed on goats and one on sheep.

## Discussion

Bluetongue has become an important animal health problem in Europe and as a result European legislation now obliges countries susceptible to the presence of BTV vectors (those located approximately between parallels 55°N and 35°S) to undertake appropriate surveillance programmes that include (i) serological surveillance, (ii) clinical inspection of livestock and (iii) entomological surveillance [18]. Here, we have confirmed (i) the presence of the potential bluetongue vector *C. obsoletus* on two of the islands in the Canary Islands archipelago (Spain) and identified, to our knowledge for the first time, its barcoding region, and have demonstrated (ii) the susceptibility of ruminants (goats and sheep) to the attacks of this biting midge species.

DNA barcoding advocates the adoption of a standard that consists of the identification of a fragment of around 650 bp of the 5´ end of the mitochondrial COI gene of each species [27]. Our results have enabled us to characterise this gene fragment for *C. obsoletus*, thereby increasing the segment previously amplified and sequenced in several studies of this particular species [12,13,28]. Using this approach, we identified two genetic haplotypes of *C. obsoletus*. Haplotype C1 was present on both islands, Gran Canaria and Tenerife, while haplotype C2 was isolated from a single biting midge captured on Gran Canaria. Our results clearly support the conclusions of previous studies and confirm the utility of molecular tools in the identification of *C. obsoletus* biting midges to species level [12].

Traditionally, studies of the feeding sources of *Culicoides* have been conducted using serological techniques [29-33] and only a few have ever investigated this feature by amplification and the sequencing of host DNA [34-36]. The cost of PCR amplification and sequencing is high; nevertheless, these tools represent an accurate way of identifying hosts to species level in studies on host-feeding patterns [37]. However, the efficacy of identification of bloodmeal sources may decrease as the stage of digestion of the host´s DNA in the abdomen of the insect increases [38]. In this respect, although we included only females with blood in their abdomen, a proportion probably had a partially digested bloodmeal whose source could not be identified. Moreover, some females may also contain a very low volume of blood, thereby reducing the success of host DNA amplification.

Both birds (mallards and common wood pigeons) and mammals (common rabbits, horses, cattle, sheep and humans) are considered potential hosts of *C. obsoletus*[34-36]. In Europe, the feeding pattern of this biting midge species was previously identified by the amplification of host DNA using mainly species-specific primers [39,40]. Our results clearly highlight the importance of ruminants, both goats and sheep, as hosts of *C. obsoletus* in the Canary Islands, a finding that could be of great importance in the transmission of diseases such as BTD. This may be the case in sheep since clinical symptoms most often manifest themselves in this ruminant [5], even though all ruminants are susceptible to infection with BTV. Further studies on the feeding patterns of this biting midge species in the Canary Islands are necessary in order to identify the susceptibility of other ruminants present in this region to attack by *C. obsoletus* females. Furthermore, the *C. obsoletus* group has also been found to be involved in the transmission of the newly emergent Schmallenberg virus [41], which affects cattle, goat and sheep production [42]. As well, the direct cost of the insect attacks that cause dermatitis in sheep has a certain veterinary importance [43].

## Conclusion

In sum, our study suggests that all the essential elements needed for an outbreak of BTD are present in the Canary Islands given that *C. obsoletus* has been shown to be one of the main vectors of this viral disease in continental Europe and given the high density of ruminants that are fed on by this midge species on these islands. Thus, the setting up of an active BTD surveillance programme on the Canary Islands is essential and sanitary laws regarding air and sea transport of potentially contaminated insects and livestock must be enforced. Nevertheless, these measures alone will not guarantee that the archipelago will remain free of the disease, since natural wind dispersal of *Culicoides* midges to the islands could occur [19,20] and as a result some infected midges could arrive from Africa.

## Competing interests

The authors declare that they have no conflicting interests.

## Authors’ contributions

Conceived and designed the experiments: JMP JM NC JF. Contributed reagents/materials/analysis tools: JMP JM MF AMN NC JF. All authors have read and approved the final manuscript.
